# Non-coding RNAs in Cardiac Intercellular Communication

**DOI:** 10.3389/fphys.2020.00738

**Published:** 2020-09-09

**Authors:** Raquel Figuinha Videira, Paula A. da Costa Martins

**Affiliations:** ^1^CARIM School for Cardiovascular Diseases, Faculty of Health, Medicine and Life Sciences, Maastricht University, Maastricht, Netherlands; ^2^Department of Molecular Genetics, Faculty of Science and Engineering, Maastricht University, Maastricht, Netherlands; ^3^Cardiovascular Research and Development Center, Faculty of Medicine, University of Porto, Porto, Portugal; ^4^Department of Physiology and Cardiothoracic Surgery, Faculty of Medicine, University of Porto, Porto, Portugal

**Keywords:** non-coding RNAs, extracellular vesicles, cardiac intercellular communication, cardiac pathological remodeling, heart failure

## Abstract

Intercellular communication allows for molecular information to be transferred from cell to cell, in order to maintain tissue or organ homeostasis. Alteration in the process due to changes, either on the vehicle or the cargo information, may contribute to pathological events, such as cardiac pathological remodeling. Extracellular vesicles (EVs), namely exosomes, are double-layer vesicles secreted by cells to mediate intercellular communication, both locally and systemically. EVs can carry different types of cargo, including non-coding RNAs (ncRNAs), which, are major regulators of physiological and pathological processes. ncRNAs transported in EVs are functionally active and trigger a cascade of processes in the recipient cells. Upon cardiac injury, exosomal ncRNAs can derive from and target different cardiac cell types to initiate cellular and molecular remodeling events such as hypertrophic growth, cardiac fibrosis, endothelial dysfunction, and inflammation, all contributing to cardiac dysfunction and, eventually, heart failure. Exosomal ncRNAs are currently accepted as crucial players in the process of cardiac pathological remodeling and alterations in their presence profile in EVs may attenuate cardiac dysfunction, suggesting that exosomal ncRNAs are potential new therapeutic targets. Here, we review the current research on the role of ncRNAs in intercellular communication, in the context of cardiac pathological remodeling.

## Introduction

Cardiac homeostasis is achieved through a complex network of interactions between the different cells of the myocardium, including cardiomyocytes, cardiac fibroblasts, neurons, cardiac endothelial, and immune cells. Upon injury, this homeostatic state is damaged and intercellular communications are rearranged toward cardiac maladaptive remodeling (Xin et al., 2013), normally characterized by cardiac hypertrophic growth, capillary rarefaction, and scar-induced events such as exacerbated inflammation, interstitial fibrosis, and ventricular dilation (Xin et al., 2013).

Cardiac communication between different types of cells can occur *via* (i) cell–matrix interactions, where cells respond to mechanical and extracellular matrix (ECM) stress; (ii) synaptic signaling, usually associated with electric and chemical neuronal signals released at the synapse site; (iii) endocrine signaling, a long distance communication where signaling factors are released into the blood stream (Kamo et al., 2015); but mostly (iv) *via* paracrine signaling, a short-range crosstalk mechanism where signals are diffused through the extracellular space before being incorporated in recipient cells and instigating a response (Bang et al., 2015). While paracrine factors have been confined for a long-time to soluble factors released from the cells, such as cytokines, in the past few years, growing evidence suggests more controlled and protected signaling mechanisms mediated by extracellular vesicles (EVs) (Hervera et al., 2019).

The classic concept of EVs containing cell debris or being markers of cell death was recently revolutionized when healthy cells were also shown to be capable of releasing these vesicles and, today, they are considered crucial mediators of both physiological and pathological cellular and molecular events. While EV is a generic term to describe a double-layer vesicle endogenously secreted by cells, their different origin, size and surface markers allow us to categorize them into three main groups: microvesicles, exosomes, and apoptotic bodies (Raposo and Stoorvogel, 2013). In contrast to microvesicles (100–1000 nm in diameter, MVs), which are formed by direct budding of the plasma membrane and, therefore, express selectins, integrins, and CD40l; exosomes (40–120 nm in diameter) originate through the cellular endocytic pathway and express markers such as CD81, CD63, CD9 (tetraspanins), flotillin, and Alix (Raposo and Stoorvogel, 2013). To date, only MVs and exosomes have been associated with intercellular communication.

Recently, a new class of EVs named exomeres has been characterized as non-encapsulated nanoparticles due to the absence of an external membrane structure, a low lipidic composition and a smaller size (up to 50 nm in diameter) (Zhang et al., 2018). The same study also distinguished two subtypes of exosomes: small exosomes (exo-S) and large exosomes (exo-L), primarily according to their size which varies from 60 to 80 nm and 90 to 120 nm, respectively. Although exo-S and exo-L present the typical exosome marker proteins Alix and Tsg101, exo-S preferentially express tetraspanins and their cargo is mainly composed of proteins associated with endosomes, multivesicular bodies, vacuoles, and phagocytic vesicles whereas Exo-L are enriched in proteins that make up the plasma membrane, cell junctions, late-endosome, and *trans-*Golgi network (Zhang et al., 2018). Given that a distinct set of proteins is found among the different exosomes, it is not surprising that they also show different degrees of stiffness and charge (Zhang et al., 2018). Due to their novelty, differences among exosomes subtypes were not taking into consideration on the studies reported in this review and, therefore, discrimination between exo-S and exo-L was not included in their reported analysis. Indeed, exosomes are generalized and referred to as a major class, without differentiation according to their subtypes, throughout this review.

**FIGURE 1:**
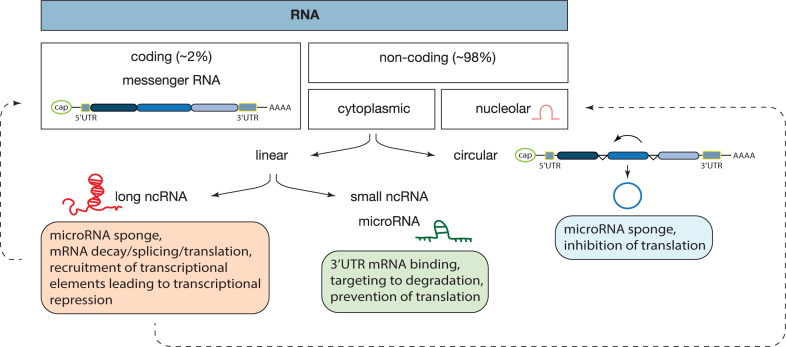
Classes of RNAs and their mechanisms of action. In our genome, approximately 98% of the transcribed RNA is ncRNA, a diverse class of RNAs that does not encode for a protein. ncRNAs are divided according to their localization: nucleus or cytoplasm; shape: circular or linear and size: small or long. Each ncRNA is a unique form and mechanism of action, as described inside of each box. In this review we focused on microRNAs, long non-coding, and circular RNAs. Dashed lines represent findings that need further validation.

Notably, exosomes can transport both proteomic and genetic cargo that are functionally active once within the recipient cell (Raposo and Stoorvogel, 2013). A great component of exosomal cargo is made up of non-coding RNAs (ncRNAs), which are major regulators of cell homeostasis and are the main players in a variety of diseases, namely cardiovascular diseases and heart failure (Dhanoa et al., 2018). ncRNAs are a heterogenous class of RNAs, classified mostly according to their size: small (miRNAs) or long (lncRNAs); shape: linear or circular (circRNAs), and cell position: nucleolar (snoRNAs) or cytoplasmic (Santosh et al., 2015; Figure 1). From bacteria to humans, ncRNAs are present in a wide range of organisms, in fact, the “baby boom” of ncRNAs occurred after the first report on the *lin-4* gene in *Caenorhabditis elegans* (Lee et al., 1993). Since then, multiple reports have implicated miRNAs in the negative regulation of gene expression at a post-transcriptional level, in biological processes ranging from cellular proliferation, migration, and apoptosis and thus, also in pathological processes such as maladaptive cardiac remodeling leading to heart failure (Liao et al., 2018; Huang S. et al., 2019; Yang et al., 2019). Typically, a miRNA selectively binds to the 3′UTR of mRNA targets and either induces their degradation or prevents them from being translated into protein, in a process that is dependent on the complementarity between the miRNA and the respective mRNA target (Li and Rana, 2014). In turn, miRNAs can be regulated by linear lncRNAs that present multiple miRNA-binding sites and which, by allowing the recruitment of several molecules at once, generate a sponge-like effect (Greene et al., 2017; Zhou and Yu, 2017). lncRNAs can also structurally facilitate mRNA decay, mRNA splicing, and translation by functioning as a dock for the “mRNA-lncRNA-Staufen-1,” a complex responsible for mRNA degradation and accumulation/assembly of specific factors of the splicing machinery by promoting the association of the mRNA 5′UTR with the polysome (Gong and Maquat, 2011; Uchida and Dimmeler, 2015). At the DNA level, lncRNAs can contribute to epigenetic modifications, namely histone and DNA methylation, by recruiting chromatin remodeling complexes such as the polycomb repressive complex 2 (PRC2) or H3K27me3 and lead to transcriptional repression (Gupta et al., 2010). In contrast to miRNAs, with their exclusive non-coding function, recent reports described a potential dual role for lncRNA as both non-coding and protein coding genes (Matsumoto et al., 2017). Recently, several lncRNAs were found to be enriched in EVs secreted by colorectal cancer cells and were shown to control gene expression patterns in the recipient cells (Hinger et al., 2018). The export of RNAs through exosomes seems to be a controlled rather than a passive or random mechanism. Despite miRNAs and lncRNAs being both incorporated in EVs, miRNAs are exported in greater quantities, and often intact, suggesting a more regulated control of exosomal miRNA content (Hinger et al., 2018).

Exosome-mediated shuttling of ncRNAs between cells is not the only mechanism of ncRNAs transference, as shown for miRNAs which can also leave the cell bound to either lipid particles such as cholesterol (Tabet et al., 2014), ribonucleic complex (Arroyo et al., 2011), and other vesicles (Chen et al., 2019). Being incorporated into vesicles grants higher miRNA longevity by conferring protection from degradation by nucleases (Arroyo et al., 2011). However, the relative abundance of cardiac secreted miRNAs within exosomes and/or other vesicles remains debatable. While Chen et al. (2020) described higher plasma levels of miR-126 in the exosomal fraction compared to the non-exosomal fraction, another study suggests that plasmatic exosomal miRNAs are only a minority, with 90% of the plasmatic miRNAs being present in a non-membrane-bound form, possibly bound to a ribonucleoprotein complex (Arroyo et al., 2011).

Similar to lncRNAs, circular RNAs (circRNAs) can also inhibit miRNAs using the “sponge” effect strategy. circRNAs are ncRNAs (exon- or intron-derived) where the 3′ and 5′ ends, usually free in a linear RNA, are linked together forming a continuous loop (Greene et al., 2017). Recently, these structurally different RNAs were associated with cardiovascular diseases and were shown to also serve as EV cargo (Zhou and Yu, 2017; Huang S. et al., 2019). In fact, an altered circRNA pattern was found in EVs derived from ischemic heart tissue when compared to EVs derived from healthy myocardium (Ge et al., 2019). The enriched circRNAs in ischemic heart tissue were associated with the metabolic process of vesicle generation while the depleted circRNAs were involved in transforming growth factor beta (TGF-β) signaling, commonly, associated with cardiac fibrosis and inflammation (Ge et al., 2019).

Here, we review the current research on the role of ncRNAs in intercellular communication, in the context of cardiac pathological remodeling.

## Cross TALK of ncRNAs in Cardiac Hypertrophy

Cardiomyocytes, the most prominent cell type of the myocardium (Zhou and Pu, 2016) can grow either *via* hyperplasia, which results in increased number of cells, or *via* hypertrophy where cell size increases while the cell number remains the same. In mammalians, adult cardiac growth occurs mainly through cardiomyocyte hypertrophy as cardiomyocytes exit the cell cycle, shortly after birth, hampering them to proliferate as an adult (Xin et al., 2013). Although physiological hypertrophic growth may be necessary to prevent cardiac atrophy during pregnancy or intense exercise, (Pu et al., 2003) hypertrophic growth is normally a maladaptive remodeling process that eventually progresses toward heart failure, depending on the nature, length, and intensity of the initial cardiac stress (Samak et al., 2016).

Intense exercise and pregnancy often result in cardiac hypertrophic growth through a process regulated by the phosphoinositide 3-kinase/protein kinase B (PI3K/AKT) signaling pathway and insulin growth factor (IGF) (Frey and Olson, 2003). As cardiac morphology and contractile function are both preserved, this is considered to be an adaptive or physiological process (Frey and Olson, 2003). In pathologic hypertrophy, where contractile function is compromised, activation of G-protein coupled receptors (GPCRs), mitogen-activated protein kinase (MAPK), and the calcineurin/nuclear factor activated cells (Cn/NFAT) pathway leads to suppression of fatty acid oxidation, increased cell growth, fibrosis, and inflammation toward heart failure (Frey and Olson, 2003). Pathological hypertrophy often develops as a consequence of chronic hypertension, valvular disease, or cardiomyopathies. Briefly, the ventricular wall is exposed to elevated mechanical stress which is perceived by the sarcomeric Z-disks in the cardiac muscle cells to trigger the activation of signaling pathways including the Cn/NFAT pathway, inducing, in turn, cardiomyocytes to become hypertrophic and ventricle walls to reach excessive dimensions (Lyon et al., 2015). Initially, an increase in size is matched with a higher heart rate as the heart tries to maintain its normal cardiac output. However, if the pathologic stimuli remains, the heart can no longer cope with the stress, and without enough oxygen due to insufficient coronary perfusion, hypertrophy becomes a maladaptive, rather than an adaptive process (Frey and Olson, 2003). In fact, the Heart Outcomes Prevention Evaluation (*HOPE*) trial, a study assessing the efficiency of ramipril, an angiotensin-converting enzyme (ACE) inhibitor, in preventing future cardiovascular events in high-risk patients, showed that inhibition or regression of cardiac hypertrophy is able to decrease heart failure and stroke events as well as increase survival rate, thus questioning whether hypertrophy is ever an adaptive process at all (Mathew et al., 2001).

Not only cardiomyocytes but also cardiac fibroblasts and endothelial cells are affected by hypertrophic stimuli, and their orchestrated response can promote cardiomyocyte growth mostly by the release of a variety of pro-hypertrophic paracrine factors. In line with this, lower levels of such factors will hamper cell growth. Inhibition of nitric oxide (NO) synthesis by endothelial cells is followed by a reduction in cardiomyocyte size (Jaba et al., 2013). Another well-described paracrine factor is TGF-β, secreted by cardiac fibroblasts. Conditioned medium of cardiac pressure overload-derived fibroblasts was shown to be sufficient to induce hypertrophic growth of cardiomyocytes in culture – an effect that could be abrogated by blockade of TGF- β receptors (Cartledge et al., 2015). These findings highlight the role of intercellular communication in the heart and its importance in the cardiac response to stress.

**FIGURE 2:**
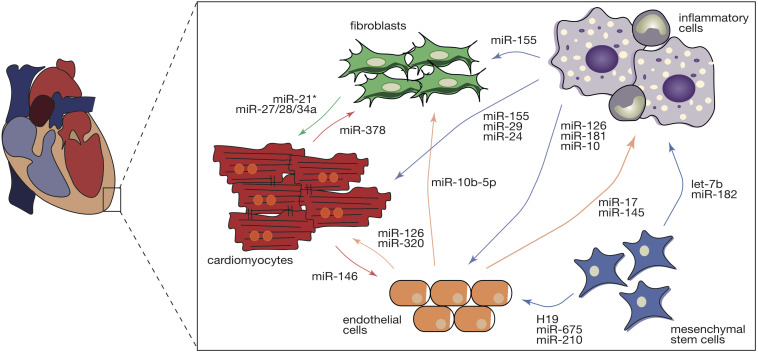
Intercellular transfer of ncRNAs during cardiac pathological remodeling. Cardiac pathological stimuli promote the various cardiac cell types to release exosomes that are enriched in several ncRNA species including miRs, circRNAs, and lncRNAs. Intercellular transfer of ncRNAs in the heart regulates diverse types of remodeling cellular and molecular processes in the recipient cells (cardiomyocytes, endothelial cells, cardiac fibroblasts, inflammatory, and mesenchymal stem cells), contributing to cardiac hypertrophy, microvascular dysfunction, capillary rarefaction, fibrosis, and inflammation. Intercellular communication is a bidirectional complex and the direction of the arrows represents the direction of the transference. Colors of the arrows symbolize the donor cell: red- cardiomyocytes, green- cardiac fibroblasts, light brown – endothelial cells, purple – inflammatory cells, and blue – mesenchymal stem cells.

Hypertrophic signaling is also influenced by the transfer of ncRNAs through crosstalk between cardiomyocytes and non-cardiomyocytes (Figure 2). Fibroblast-derived exosomes were shown to be enriched in miR-21^∗^ which can be taken up by cardiomyocytes and can induce their hypertrophic growth by targeting cytoskeletal proteins, namely sorbin and SH3 domain-containing protein 2 (SORBS2) and PDZ and LIM domain 5 (PDLIM5) (Tian et al., 2018). In line with this, blocking of miR-21^∗^ in mice subjected to chronic administration of angiotensin II (AngII) reduced cardiomyocyte growth, underlining fibroblast-derived miR-21^∗^ as a key player toward a pathologic cardiac response to chronic stress (Tian et al., 2018). More recently, miR-34a, along with miR-27a and miR-28a were reported to be preferentially incorporated into exosomes secreted by cardiac fibroblasts after tumor necrosis factor alpha (TNF-α) treatment (Tian et al., 2018). These miRNAs are secreted from cardiac fibroblasts and transferred to cardiomyocytes where they not only induce expression of hypertrophic genes but also have as a common target an antioxidant enzyme, Kelch-like ECH-associated protein 1-nuclear factor erythroid 2-related factor 2 (Nrf2), previously shown to be downregulated in chronic heart failure (Tian et al., 2018). By being upregulated in chronic heart failure, miR-27a, miR-28a, and miR-34a may contribute to the development of the disease through the inhibition of Nrf2 (Tian et al., 2018).

Similarly, macrophages are also able to influence the cardiomyocyte response to stress by transferring macrophage-derived miR-155 (Halkein et al., 2013). *In vitro*, the hypertrophic phenotype of cardiomyocytes treated with the pro-hypertrophic factor endothelin 1 (ET1) was attenuated by exposing the cells to conditioned media derived from miR-155-deficient macrophages (Wang C. et al., 2017). *In vivo*, specific genetic ablation of miR-155 on macrophages hampered cardiac hypertrophy and inflammation in a mouse model of hypertrophy (Wang C. et al., 2017). Regarding endothelial cells, it was shown that in peripartum cardiomyopathy, a cardiac disease initiated by a cleaved prolactin fragment (16-kDa N-terminal prolactin fragment, 16K PRL), increased expression of miR-146 hampering angiogenesis (Halkein et al., 2013). However, miR-146 was also found in endothelial cell-derived exosomes and was shown to be transferred to cardiomyocytes where it negatively regulates Erb-B2 Receptor Tyrosine Kinase 4 (*Erbb4*), known to be involved in cardiac hypertrophy and metabolism. Chemical inhibition of miR-146 in a mouse model of peripartum cardiomyopathy attenuated cardiac dysfunction (Halkein et al., 2013).

Cardiac hypertrophy, a fundamental risk factor for heart failure, develops as an orchestrated response of several types of cells where ncRNAs act as paracrine factors manipulating the behavior of both donor and recipient cells (Figure 2).

## Cross TALK of ncRNAs in Cardiac Capillary Rarefaction

As shown for several vascular diseases such as atherosclerosis, vessel restenosis, and primary hypertension, the vasculature is mostly capable of adapting itself in response to different stimuli to maintain vascular function and structure after injury (Su et al., 2017). However, pathologic stimulation of the cardiac vessels results in increased rigidity and a decline of vessel compliance as maladaptive vascular remodeling that will ultimately further contribute to cardiomyocyte hypertrophy and fibrosis (Su et al., 2017). Larger cardiomyocytes require more oxygen and nutrients and to cope with this increased demand, the cardiac vasculature must expand accordingly. In an initial phase, proangiogenic signals are released from damaged cardiomyocytes and capillaries are able to adapt to the increased demand, however, if the stress persists leading to exaggerated hypertrophy, these mechanisms fail to further stimulate vascular growth (Gogiraju et al., 2019). In pressure overload-induced hypertrophy, an increase in p53 expression leads to apoptosis of endothelial cells, deficient vasculature, and diminished capillary density – all contributing to tissue hypoxia and subsequent scar formation (Gogiraju et al., 2019).

One of the mainstays of cardiac vascularization and function is the vascular endothelial growth factor (VEGF) signaling pathway. Constitutive expression of VEGF-A in mice is able to enhance cardiac angiogenesis and improve basal cardiac function and *vice versa*, while its depletion results in defective vascularization, thinner cardiac walls, and contractile dysfunction (Lee et al., 2017). The VEGF signaling is also under the control of miRNAs including miR-374, shown to target VEGF receptor 1 (VEGFR1) and its downstream factor protein kinase G1 (PKG1), a regulator of calcium release from endoplasmatic reticulum that, indirectly, promotes hypertrophy (Lee et al., 2017). Depletion of miR-126, another miRNA regulating angiogenesis, in endothelial cells leads to partial embryonic lethality (40%) due to vascular defects (Wang et al., 2008). Similarly, in mice subjected to myocardial infarction (MI), miR-126 deletion contributed to a decrease in the VEGF signaling pathway (Yang et al., 2017). Following MI, the ischemic myocardium responds by increasing VEGF and fibroblast growth factor (FGF) expression, two potent angiogenic factors that are crucial for the development of collateral vessels and contribute to the reperfusion of the damaged myocardium (Cochain et al., 2013). MiR-126 silencing promotes the expression of its target Sprouty Related EVH1 Domain Containing 1 (SPRED1), an inhibitor of the RAF proto-oncogene serine/threonine-protein kinase (RAF1) and VEGF/ERK angiogenic pathway (Fish et al., 2008). Interestingly, miR-126 was reported to be depleted in exosomes derived from type 2 diabetic rat cardiomyocytes, while miR-320 was found to be highly enriched (Wang et al., 2014). Uptake of these miR-320-enriched exosomes by cardiac endothelial cells compromised their proliferative, migratory, and tube formation capacity (Wang et al., 2014). In contrast, inhibition of miR-320 or pharmacological inhibition of exosome formation were sufficient to abrogate the deleterious effects of miR-320 expression on angiogenesis – indicative of an anti-angiogenic and exosome-dependent role for this miRNA (Wang et al., 2014).

Moreover, depletion of miR-17 and miR-145 from exosomes derived from macrophages that were exposed to AngII, interferes with the capacity of cells to incorporate those exosomes (Leisegang et al., 2017). In endothelial cells, exosomal depletion of miR-17 and miR-145 increased the expression of two previously described targets known to be involved in inflammation, intercellular adhesion molecule 1 (ICAM-1), and plasminogen activator inhibitor 1 (PAI-1) (Osada-Oka et al., 2017). These results underline miRNAs as pathological cargo of exosomes, emphasizing their role in promoting pro-inflammatory signaling pathways in endothelial cells.

Similar to miRNAs, lncRNAs can also act as regulators of angiogenesis. CRISPR/Cas9-mediated genetic deletion of a lncRNA that is normally found upregulated in vascular pathologies (Leisegang et al., 2017), *MANTIS*, in human umbilical vein endothelial cells (HUVECs), compromised their tube formation and sprouting capacity through the downregulation of several angiogenic-related genes (Leisegang et al., 2017).

Interestingly, exposing neonatal rat cardiomyocytes to hypoxic conditions induced the release of exosomes that are enriched in circRNAs, namely, circHIPK3 (Wang et al., 2019b). Conditioned media derived from hypoxic cardiomyocytes led to upregulation of this circRNA in cardiac microvascular endothelial cells (CMVECs). In contrast, circHIPK3 is downregulated when CMVECs are exposed to H_2_O_2_. In these cells, circHIPK3 acts as a sponge for miR-29, that in turn, targets the insulin-like growth factor 1 (IGF-1) pathway, involved in reactive oxygen species (ROS) production and apoptosis (Yanagisawa-Miwa et al., 1992). In fact, re-expression of IGF-1, due to enrichment of circHIPK3, promoted survival of CMVECs exposed to H_2_O_2_ and decreased ROS production, suggesting that cardiomyocyte exosome-derived circHIPK3 is a regulator of endothelial function and capillary rarefaction during hypoxic conditions (Wang et al., 2019b).

A defective vasculature is typical of the maladaptive cardiac remodeling process observed in different pathologies, including MI, hypertension, or cardiomyopathy (Cochain et al., 2013). Increased capillary density after cardiac injury has been shown to prevent cardiac remodeling and improve cardiac function, establishing angiogenesis as an appealing therapeutic target (Yanagisawa-Miwa et al., 1992). Besides being effective regulators of angiogenesis, incorporation of ncRNAs into exosomes protects them and facilitates their intercellular transfer, making them attractive tools for angiogenic therapy (Huang P. et al., 2019).

## CrossTALK of ncRNAs in Cardiac Fibrosis

The very limited regenerative capacity of the mammalian adult heart due to low proliferative capacity and defective angiogenesis leads to the formation of a permanent scar after ischemic injury (Xin et al., 2013). Cardiac fibrosis is the main cellular event leading to scar formation and is characterized by an accumulation of ECM in the myocardium (Beltrami et al., 2003). In healthy conditions, ECM is responsible for transmission of the contractile force and serves as scaffolding for cardiac cells conferring mechanical support to the heart, and as such, cardiac contraction and relaxation firmly depends on ECM (Michel, 2003; Bildyug, 2019). Cardiac ECM is predominantly composed of collagens I and III (ColI and ColIII), glycosaminoglycans, glycoproteins, latent growth factors, and proteases (Kong et al., 2014), and is under constant turnover of sustained degradation and synthesis processes (Lajiness and Conway, 2014). Dysregulation of this tightly controlled process may impair both systolic and diastolic function as result of uncoordinated cardiomyocyte contraction, and/or cardiomyocyte displacement associated with ventricular dilation, and, ultimately cause ventricular stiffness and arrhythmias. The hallmarks of the cardiac fibrotic response include activation of fibrillary collagen and myofibroblast differentiation from either fibroblasts, hematopoietic, or endothelial cells (Davis and Molkentin, 2014). Myofibroblasts display contractile stress fibers and a large endoplasmic reticulum – associated with secretion of large amounts of ECM proteins – and are very sensitive to growth factors and pro-inflammatory molecules (Davis and Molkentin, 2014). Despite being essential for myocardium repair, continuous presence of myofibroblasts in response to persistent inflammatory signals may result in excessive scarring and impairment of both systolic and diastolic function (Davis and Molkentin, 2014).

The molecular mechanisms underlying cardiac fibrosis are very dependent on the type of primary myocardial injury; however, there are fibrogenic signals consistently activated during fibrosis such as increased levels of TGF-β and AngII, among several others (Kong et al., 2014). TGF-β is a cytokine present as three different isoforms (TGF-β1, 2, and 3) and, while in the heart TGF-β1 is mainly present as a latent complex, once it is activated upon injury through the matrix metalloproteases 9 and 2 (MMP-9, MMP-2), it will cause alterations in the ECM composition (Ignotz and Massague, 1986). While overexpression of TGF-β1 promotes collagen deposition and ventricular fibrosis, its loss of function attenuates myocardial fibrosis in a rat model of pressure-overload (Koitabashi et al., 2011).

Several studies have indicated cardiac fibrosis to be under control of ncRNAs. In fact, TGF-β1 activity is regulated by miR-21, which by targeting Jagged1, a ligand for Notch receptor 1, affects *trans-*differentiation of cardiac fibroblasts into myofibroblasts and subsequently, myocardial fibrosis (Zhou et al., 2018).

Global lncRNA expression profiling in cardiac fibroblasts revealed lncRNA *Meg3* to be specifically enriched in cardiac fibroblasts (Piccoli et al., 2017). Upon pressure overload, *Meg3* regulates MMP-2 expression by stabilizing p53 at its binding site on the MMP-2 promoter, resulting in cardiac fibrosis and diastolic dysfunction (Piccoli et al., 2017). Silencing of lncRNA *Meg3* with gapmers, prevented MMP2 activation and had a protective effect in pressure-overloaded hearts, partially due to attenuation of fibrosis (Piccoli et al., 2017). Given the emerging importance of circRNAs in myocardial function, Zhou and Yu (2017) analyzed the circRNA expression pattern in myocardial tissue of diabetic mice. A circRNA microarray screening identified circRNA_010567 as one of the top upregulated circRNAs in cardiac fibroblasts and its inhibition in cardiac fibroblasts exposed to AngII suppressed fibrosis-associated genes such as *Col I, Col III, and* alpha smooth muscle actin (α*-SMA)* (Zhou and Yu, 2017). circRNA_010567 was shown to have multiple binding sites for miR-141, that in turn targets TGF-β1. In fact, silencing of circRNA_010567, increased miR-141 expression, decreased TGF-β1 expression and its downstream targets, attenuating myocardial fibrosis (Zhou and Yu, 2017).

Apart from fibroblast-derived ncRNAs, there are other ncRNAs that originate from different cell types and are implicated in cardiac fibrosis through paracrine signaling. A recent study demonstrated that mechanical stress due to pressure-overload promotes transfer of cardiomyocyte-derived miR-378 to cardiac fibroblasts in an EV-dependent manner (Yuan et al., 2018). Loss- and gain-of function of miR-378 in cardiomyocytes, *via* targeting of the TGF-β1 pathway, accentuated collagen deposition but inhibited cardiac fibroblast proliferation. Similar effects were observed when EV secretion was impaired by administration of a sphingomyelin phosphodiesterase inhibitor, GW4869 (Yuan et al., 2018), suggesting that the mechanism used by cardiomyocytes to release miR-378 and to exert its function in cardiac fibroblasts, is dependent on intercellular communication *via* exosomes.

As mentioned, myofibroblast activation is a key contributor to cardiac fibrosis and cardiac pathological remodeling, and the transition between fibroblasts and myofibroblast can be regulated by exosomal miRNAs, namely, cardiomyocyte-derived miR-195 (Morelli et al., 2019). Upon cardiac ischemic injury, cardiomyocytes release exosomes that are enriched in miR-195. *In vitro* exposure of cardiac fibroblasts to conditioned medium derived from myocardial infarction (MI)-hypoxic cardiomyocytes, led to an increase in the levels of miR-195, as well as in myofibroblast and fibrotic markers such as periostatin, alpha smooth muscle actin (α-SMA), and ColI and III on cardiac fibroblasts. Mechanistically, miR-195 targets the α-SMA inhibitor mothers against decapentaplegic homolog 7 (SMAD7), promoting the transcription of α-SMA and other myofibroblast associated genes (Morelli et al., 2019). Inhibition of EV release or inhibition of miR-195, *via* administration of the GW4869 or miR-195 inhibitor, respectively, abrogated myofibroblast activation. These observations established miR-195 as an important player during MI-induced pathological remodeling through myocyte-fibroblast communication (Morelli et al., 2019).

Macrophages, in turn, are able to affect cardiac fibrosis by transferring miR-155 into cardiac fibroblasts (Wang C. et al., 2017). An increment in exosome secretion by macrophages is accompanied by an increase in miR-155 expression in cardiac fibroblasts while deletion of miR-155 on macrophages leads to a reduction of miR-155 levels in cardiac fibroblasts. However, abrogation of miR-155 expression in cardiac fibroblasts did not interfere with their endogenous levels of miR-155, suggesting that the origin of miR-155 in cardiac fibroblasts derives from an external source, namely, macrophages. In cardiac fibroblasts, increased miR-155 expression prevents cell proliferation and increases inflammation by targeting fibrotic and inflammatory genes, simultaneously. Genetic deletion of miR-155 *in vivo* attenuated inflammation but accelerated cardiac fibroblast proliferation after MI, inhibiting myocardial rupture and improving cardiac function (Wang C. et al., 2017).

While the concept of exosomal miRNAs in myocardial fibrosis is well established, implication of exosomal lncRNAs and/or exosomal circRNAs transfer during cardiac fibrosis were still not reported, highlighting the need for better understanding of the molecular mechanisms driving cardiac fibrosis as a dichotomous event, in order to pursue more efficient therapeutic targets.

## CrossTALK of ncRNAs in Cardiac Inflammation

Inflammation is a common ground for maladaptive cardiac remodeling, independent of the disease etiology. Inflammation occurs in all stages of tissue repair including initiation, maintenance, resolution, and clearing out the damaged tissue. A vast variety of cells, including monocytes, macrophages, dendritic cells, T-cells, B-cells, NK cells, and others, compose the immune system and regulate the inflammatory response. Both MI and hypertension activate an inflammatory cardiac response; however, the mechanisms induced by these two types of injury are very distinct.

In MI, inflammation is initiated following the typical hallmarks of ischemic injury, cardiomyocyte death, and interstitial fibrosis, both involved in the generation of reactive oxidative species and activation of the complement pathway (Prabhu and Frangogiannis, 2016). Subsequently, endothelial dysfunction leads to increased vessel permeability followed by leukocyte infiltration. Finally, remodeling of the ECM, along with damaged cardiac cells’ release of interleukins (IL) like IL one alpha (IL-1α) and heat shock proteins (HSP) that act as damage-associated molecular patterns (DAMPs), takes place (Prabhu and Frangogiannis, 2016). When secreted to the extracellular environment these danger signals bind to pattern recognition receptors activating leukocytes (Prabhu and Frangogiannis, 2016). Hearts subjected to pressure overload either by hypertension or aortic stenosis, show a profound vascular remodeling accompanied by perivascular fibrosis and inflammation but no significant cardiomyocyte loss (Xia et al., 2009). Cardiac hypertrophy is followed by transient activation of the inflammatory response, starting with the release of pro-inflammatory cytokines such as IL-1β, IL-6, and TNF-α and rapidly progressing to macrophage infiltration and upregulation of TGF-β. Long term inflammation is replaced by fibrosis and the initial cardiac response develops into both diastolic and systolic dysfunction (Xia et al., 2009).

Macrophages display different inflammatory states. Usually, M1 macrophages are associated with a pro-inflammatory phenotype by releasing IL-1β, IL-6, and TNF-α, while M2 phenotype transition is linked to immunomodulation and secretion of anti-inflammatory cytokines such as IL-10. Notably, M1 to M2 macrophage transition might be influenced by bone marrow-derived mesenchymal stem cells (BMSCs)-derived exosomes (Xu et al., 2019) as myocardial injection of such exosomes in the border zone of ischemic hearts, significantly decreased M1/M2 ratio and attenuated cardiac inflammation. These protective effects were enhanced by exosomes derived from BMSCs after preconditioning with lipopolysaccharide (LPS) stimulation (Xu et al., 2019). Similar to BMSCs, LPS preconditioning of mesenchymal stromal cells can also guide M1 to M2 macrophage activation along with the upregulation of anti-inflammatory cytokines through exosomal release (Ti et al., 2015). Interestingly, exosomes derived from LPS-activated MSCs are enriched with let-7b which regulates macrophage plasticity *via* toll-like receptor 4 (TLR4), and *in vivo*, increases diabetic cutaneous wound healing and attenuates inflammation (Ti et al., 2015). Another report on mesenchymal stromal cells-derived exosomes highlighted the cardioprotective and immunomodulatory effect of exosomal miRNAs on M1/M2 macrophage shuttle (Zhao et al., 2019). Under inflammatory conditions, such as myocardial ischemia/reperfusion, miR-182 is loaded in MSCs-derived exosomes and further incorporated into macrophages (Zhao et al., 2019). Similar to let-7b, miR-182 also targets the TLR4 pathway and activates the PI3K/AKT signaling pathway, an important step in the conversion of M1 into anti-inflammatory M2 phenotype (Zhao et al., 2019).

Reduction of the inflammatory process and improved immunomodulatory response were shown upon addition of endothelial cell-derived EV to monocytes (Njock et al., 2015). Endothelial cells exert their anti-inflammatory effect by secreting EVs loaded with miR-126, miR-181b, and miR-10. Once taken up by monocytes, miR-10 induced the strongest anti-inflammatory effect by repressing the nuclear factor κB (NF-κB) pathway and increasing the levels of immunomodulatory genes (Njock et al., 2015). This supports an essential role of the crosstalk between different cell types such as endothelial cells and monocytes, during cardiac inflammation cardiac, mediated by miRNAs enclosed in secreted EVs.

## Inter-Organ Crosstalk

**FIGURE 3:**
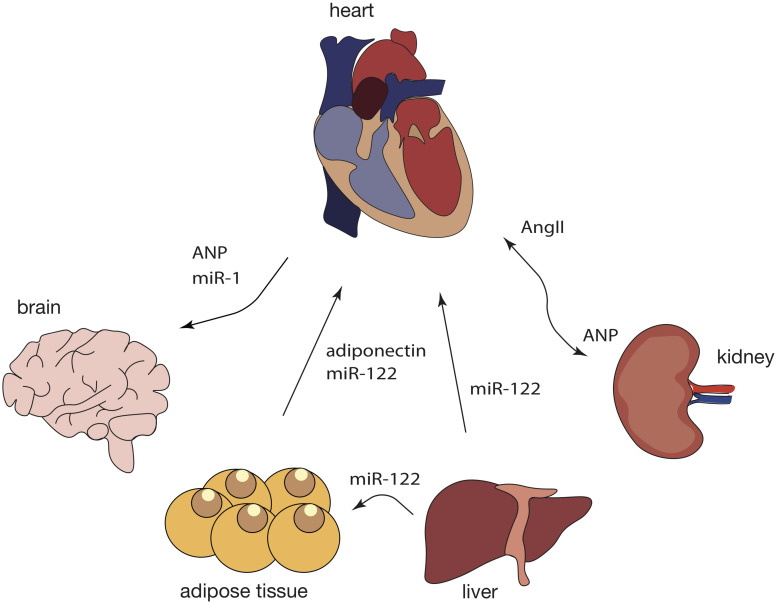
Crosstalk between the heart and peripheral organs during cardiac pathological remodeling. Response to cardiac injury is a multi-organ process as it affects not only the heart but also the brain, kidneys, adipose tissue, and the liver, among others. In turn, a variety of other tissues can contribute to cardiac pathological remodeling, not only by secreting specific chemokines but also by releasing exosomal ncRNAs into the blood stream and further affecting cardiac function through intricate signaling networks. Dashed lines represent findings that need further validation.

The primary function of the heart is to pump blood to the rest of the body, yet, recent studies demonstrate that the heart could have other functions and acts as an endocrine organ (de Bold et al., 1981; Nakamura et al., 1991). Through endocrine signaling, the heart is able to maintain whole body homeostasis and, in this way, peripheral organs are able to respond to stress stimuli that originate in the heart (Figure 3).

Most likely, the best described cardiac endocrine factor is the atrial natriuretic peptide (ANP), the expression of which is increased after myocardial stretching (Hardy-Rando and Fernandez-Patron, 2019). A previous study reported that upon corticosterone stimulation, astrocytes release “ANPergic vesicles” (vesicles enriched in ANP) (Chatterjee and Sikdar, 2013). In the brain, ANP acts as an inhibitory signal that regulates astrocyte mobility, neuron signaling and blood flow to the brain due to its vasoregulatory action (Chatterjee and Sikdar, 2013). Although no evidence of ANP vesicles has been reported in the heart, it is possible that ANP-containing vesicles constitutes a mechanism of ANP transport and action within the cardiovascular system, as well as modulators of heart-kidney communication and particularly, blood pressure-control.

Atrial natriuretic peptide can influence and suppress the sympathetic activity in the heart through a decrease in the activity of chemo and baroreceptors, which leads to lower heart rate and cardiac output (McGrath et al., 2005). Since adipose tissue presents ANP receptors, the release of ANP by the heart, can trigger lipolysis, energy expenditure and shifting of adipokine production and release (Gruden et al., 2014). In mice subjected to MI, miR-214, typically enriched in adipose-derived regenerative cells (ADRCs), is transferred into cardiomyocytes, where it suppresses apoptotic gene expression such as Bcl-2-like protein 11 (*Bcl2l11*) and solute carrier family 8 member A1 (*Slc8a1*), preventing cardiac rupture (Eguchi et al., 2019). Cardiomyocytes can also uptake miR-214 derived from ADRCs, suggesting that EVs containing miR-214 are endocytosed through a clathrin-mediated endocytosis process triggered by MI-induced hypoxia (Eguchi et al., 2019).

However, the opposite can also occur. For example, in obesity, metabolic stress induces changes in adipose tissue, and increased secretion of adipokines is perceived by the heart and impacts on its function. One of these adipokines, adiponectin, is decrease in several cardiovascular diseases, including MI (Shojaie et al., 2009), and hypertension (Wang et al., 2012; Brambilla et al., 2013). In cardiomyocytes, adiponectin is able to activate the adenosine monophosphate-activated protein kinase signaling pathway inhibiting pressure-overload induced hypertrophy (Turdi et al., 2011).

An increase in heart rate and contractility is regulated by the sympathetic system, namely, postganglionic efferent neurons that communicate with cardiomyocytes by releasing noradrenaline and cause the activation of β-adrenergic receptors (Bang et al., 2015). In contrast, heart rate decrease is under the control of the parasympathetic system *via* acetylcholine, which binds to muscarinic receptors, and dysregulation of these hormones could lead to arrhythmias (Bang et al., 2015).

Besides the adipose tissue and the brain, the heart also communicates with the kidneys through the renin-angiotensin system (RAS), mainly associated with regulation of blood pressure (Jahng et al., 2016). In a healthy heart, low blood pressure stimulates the kidneys to produce renin, which, in turn, leads to increased AngII, followed by sodium and water retention. Finally, an increase in blood volume and increased pressure stimulate cardiomyocytes to release ANP into the circulation, through which it will reach the kidneys (Song et al., 2015). Here, ANP activates excretion of salts and water reducing both blood volume and pressure. In hypertension, this system is overly activated and the heart is not able to decrease the blood pressure, causing continuous secretion of ANP and subsequently maladaptive cardiac remodeling (Orsborne et al., 2017).

Interestingly, not only proteins participate in cardiac inter-organ communication. In obese mice, cardiac injury can be induced by liver-derived exosomal miR-122 (Wang et al., 2019a). MiR-122 is liver-specific and, upon accumulation of visceral fat, miR-122 is released in liver-derived exosomes and transported to the heart, where it interferes with mitochondrial adenosine triphosphate (ATP) production, and eventually, left ventricle (LV) function (Wang et al., 2019a). Deletion of hepatic miR-122 significantly improved LV function parameters in obese mice (Wang et al., 2019a). This miRNA, by also being enriched in plasmatic exosomes from obese humans, is a promising target against obese-induced cardiac injury.

As discussed before, the heart can also send signals to other organs, including the brain. Upon MI, cardiomyocytes increase the release of exosomes enriched in miR-1 (Sun et al., 2018). These exosomes are carried through the blood flow and incorporated by the hippocampus to induce neuronal microtubule damage (Sun et al., 2018). Genetic ablation of cardiomyocyte-derived miR-1 significantly attenuated neuronal damage in mice subjected to MI, suggesting that miR-1 acts as an endocrine factor (Sun et al., 2018).

To date no other classes of ncRNAs have been reported to have an endocrine effect to or from the heart.

## Therapeutic Potential of Cardiac Exosomal ncRNAs

Exosomal ncRNAs constitute a new form of paracrine signaling that seems to be more protected, directed, and therefore, most likely also more efficient. Naturally, exosomes are endogenously produced by cells, but systemic isolation of exosomes that are secreted by one specific cell type is still difficult. Therefore, all the studies reported in this review are based on exosomes that were produced *in vitro* and then administrated *in vivo*. Consequently, these cell culture-derived exosomes are of exogenous origin, relative to the recipient mouse, which, in turn are also able to naturally produce endogenous exosomes. These exogenous exosomes may have an enormous therapeutic potential. In fact, exosomes revolutionized the delivery strategies of therapeutics as the use of these vectors in cell-free medicine will, most likely, overcome the barriers associated with cell transplantation such as contamination and cell death (Toma et al., 2002) and decrease the risk of tumorigenicity and immune rejection (Chen Y. et al., 2017). The smaller size of exosomes allows immediate and easy circulation through capillaries, opposed to what was observed for cell-based therapies such as mesenchymal stem cell (MSC) transplantation, where many cells do not manage to pass the first capillary bed (Phinney and Pittenger, 2017). Exosomes can potentially carry the same miRNA profile of the donor cell, which may potentiate the beneficial effects observed in traditional cell transplantation (Shao et al., 2017). Likewise, in a mouse model of MI it was shown that the protective effect of MSC transplantation was promoted by exosomal miR-125, which, by targeting p53 reduced the apoptotic flux and, consequently, cell death, while its deletion abrogated these effects (Xiao et al., 2018). Transplantation of MSC-derived exosomes alone, revealed to be sufficient to recapitulate MSC-associated beneficial effects. Furthermore, exosomal ncRNA content can be, partially, modulated by transfecting donor cells with a specific ncRNA mimic or inhibitor (Xiao et al., 2018) that will be, in turn, naturally released in exosomes, improving therapy efficacy, as demonstrated by Zhu et al. (2019). The presence of lncRNA *MALAT-1* in exosomes derived from umbilical cord MSCs is able to alter NF-κB signaling and exert cardioprotection in an *in vitro* model of aging (Zhu et al., 2019). The facility in obtaining MSCs, isolating their released exosomes and modeling their miRNA content, are particularly attractive features for their therapeutic use.

In fact, MSCs, have been extensively studied as therapeutic targets for MI, due to their reparative capacity (Przybyt and Harmsen, 2013; Chen L. et al., 2017; Luger et al., 2017). Since transplantation of MSCs has high risks, a therapy based on EVs could be advantageous. Analysis of the MSC-derived exosome contents revealed enrichment of miR-24 and miR-29 and depletion of miR-130, miR-378, and miR-34 (Shao et al., 2017). While the enriched miRNAs positively associate with the regulation of cardiac function, the depleted miRNAs were negative regulators. Interestingly, addition of MSCs-derived exosomes to cardiomyocyte cultures was sufficient to stimulate cardiomyocyte proliferation (Shao et al., 2017). Another study reported that hypoxic preconditioning enhances the protective effect of MSCs, manifested by decreased scar size and improved cardiac function after MI, through exosome-mediated signaling (Zhu et al., 2018). Exosomes secreted by MSCs can be incorporated into cardiac endothelial cells, increase their angiogenic capacity and, ultimately, improve cardiac function (Zhu et al., 2018). MSC-derived exosomes are also enriched in miR-210, known as the “master hypoxamir,” (Chan et al., 2012) which overexpression promotes to tube formation but its depletion fails to promote angiogenesis on endothelial cells (Zhu et al., 2018). Furthermore, Wang K. et al. (2017) unraveled that the mechanism whereby endometrium-derived MSCs (EndMSC) can exert their cardiac protective effect is by exosomal release of miRNAs, namely, miR-21. EndMSC secrete miR-21 that is shuttled to both cardiomyocytes and endothelial cells, preventing apoptosis and promoting angiogenesis after MI, respectively (Wang K. et al., 2017).

In turn, lncRNA *H19* was shown to have a paracrine effect upon atorvastatin (AT)-conditioned (MSC) treatment, as AT treatment induced MSCs to produce exosomes that were enriched in lncRNA H19 and miR-675, that once internalized by endothelial cells enhanced their migratory, proliferative and sprouting capacity and therefore, angiogenesis (Huang P. et al., 2019). In fact, in a mouse model of MI, administration of H19-enriched, MSC-derived exosomes, significantly reduced infarct size and increased left ventricle ejection fraction (Huang P. et al., 2019).

Due to their high proliferative capacity, endothelial colony forming-cells (ECFCs), progenitor of endothelial cells, are able to reduce fibrosis after MI, making them an attractive target for cell-based therapies, even though their beneficial effect is hampered under hypoxic conditions (Liu et al., 2018). ECFCs have shown to enhance vascularization by stabilizing the perivascular area and secretion of angiogenic molecules (e.g., VEGF-A), to attenuate fibrosis, and to increase cardiomyocyte survival possibly due to increased expression of IGF-1, an anti-apoptotic factor (Liu et al., 2018). ECFC-derived exosomes are thought to be the mediators of the observed protective effects. Adding exosomes derived from ECFCs exposed to normoxia to cardiac fibroblast cultures, significantly reduced fibroblast activation, TGF-β stimulation, and the fibroblast expression levels of *Col-I*α and α*-SMA* (Liu et al., 2018). Exposing cardiac fibroblasts to hypoxic ECFCs-derived exosomes increased fibrosis and showed a similar effect to treatment of TGF-β alone. miRNA content analysis revealed miR-10b-5p as the most enriched miRNA in normoxia ECFCs-derived exosomes when compared to hypoxia ECFCs-derived exosomes, which, curiously, targets the downstream factors of the TGF-β pathway, SMAD ubiquitylation regulatory factor 1 (SMURF1) and histone deacetylase 4 (HDAC4) (Liu et al., 2018).

A study reported that intravenously and intraperitoneal injections of exogenous exosomes does not induce toxicity (no body weight or behavioral changes) nor an alteration of hematological and biochemical parameters (Zhu et al., 2017). As EVs were only detectable at low levels in the spleen and not detectable at all in the liver or spleen, this indeed suggests that there is no toxicity or severe immune response (Zhu et al., 2017). Nevertheless, not only is more research needed to draw a conclusion on exosome cardiotoxicity, a better understanding of miRNA off-targets effects is also necessary before exomiRs reach clinical practice.

## Conclusion

Intercellular communication in the heart contributes to both the maintenance of cardiac function and the development of cardiac pathologies, characterized by cardiac hypertrophy, fibrosis, inflammation, and deficient vasculature. Communication between cardiomyocytes, fibroblasts, inflammatory cells, and endothelial cells in the heart is not unidirectional but bi/multidirectional, as each cell type profoundly influences each other’s behavior. Most of the mechanisms driving this cell-to-cell crosstalk are relatively unknown, thus, analyzing the changes in cell signaling upon a pathological stimulus may yield new information to prevent or reverse pathological remodeling. Although intercellular communication can occur in a variety of forms, here we focused on the paracrine signaling through EVs, namely, exosomes. Many reports have been focused on how the modulation of ncRNAs can successfully prevent and even reverse cardiac maladaptive remodeling. Despite the promising value of several miRNAs, lncRNAs, and circRNAs, as new therapeutic targets, to date, miR-122 is the only ncRNA that has reached a phase II clinical trial.

Interestingly, ncRNAs can be loaded into EVs and further incorporated into others cell types, where they are still capable of efficiently exert their action and consequently, trigger a response. However, since the majority of the current studies were based on the isolation of vesicles from cellular cultures, there is a considerable gap in the *in vivo* EV characterization, as well as in understanding their spatial and temporal secretion in the heart. Furthermore, exosomal ncRNAs have an unpredictable toxicity when administrated *in vivo* due to the short-observation span of the majority of the studies. Finally, we believe that understanding and mimicking the action of EVs, by modifying EV ncRNA content, will constitute a future therapeutic target.

## Author Contributions

RV and PC wrote the manuscript and designed the figures. PC revised the manuscript. Both authors contributed to the article and approved the submitted version.

## Conflict of Interest

PC is a cofounder of Mirabilis Therapeutics. The remaining author declares that the research was conducted in the absence of any commercial or financial relationships that could be construed as a potential conflict of interest.
